# The Greatest Loss

**Published:** 1870-07

**Authors:** 


### THE GREATEST LOSS

Is the loss of temper; it unfits us for the social circle ; it unfits for business, for pleasure and for religion. The exhibition of a loss of temper always degrades; the man himself feels it a very few moments afterwards.

A very frequent cause of an irritable disposition, of a fretful, complaining temper, always ready to oppose, object and find fault, is dyspeptic indigestion. A person at the table will be gentle, generous, entertaining and agreeable; an hour later, and the whole character is changed; there is a querulousness, a rudeness, a sulkiness, a contempt of every rule of courtesy and politeness, which is as amazing as it is degrading. It is the healthy man who is

THE SAME ALL THE TIME, who always meets you with a cordial welcome and a cheerful manner. You are never sure in approaching a dyspeptic, even though your nearest and best friend, that you will not be met with a snap or a snarl; some spiteful remark or some ill-natured action. To keep your temper then, keep well; make the preservation of your health a study and a duty; it will pay handsomely; a cheerful heart makes the hands nimble and the brain active, keen, ever on the alert; hence, good health is an important element of business success; and more than that, the greatest of modern preachers, the most successful, as also those of a past age, were and are among the healthy men; giving them high animal spirits, as well as bodily vigor and mental elasticity, and wit and fun, and a certain degree of impudence, fearlessness and self-assertion. It is refreshing to see a man go into the pulpit with a certain degree of force and fearlessness; ready to do and dare anything that becomes his office; as if he felt himself inferior to none, as a man; superior to all, in his office.

Some men go into the pulpit as if they were afraid to speak; as if they were afraid they would break in two; as if they were a sheep, so meek are they, so prim and proper. A queen once looked down into the grave of a minister and said, " There lies he who never feared the face of man." It was John Knox. But, reader, the manner of John Knox must have his mind to go with it, or you will make a fool of yourself.

